# Generation of motor neurons from human amygdala-derived neural stem-like cells

**DOI:** 10.22038/IJBMS.2018.29587.7146

**Published:** 2018-11

**Authors:** Sepideh Ghasemi, Hadi Aligholi, Pir Hossein Koulivand, Maryam Jafarian, Hassan Hosseini Ravandi, Maryam Khaleghi Ghadiri, Ali Gorji

**Affiliations:** 1 Shefa Neuroscience Research Center, Khatam Alanbia Hospital, Tehran, Iran; 2 Department of Neuroscience, School of Advanced Medical Sciences and Technologies, Shiraz University of Medical Sciences, Shiraz, Iran; 3 Department of Neurosurgery, Westfälische Wilhelms-Universität Münster, Münster, Germany; 4 Department of Neuroscience, Faculty of Medicine, Mashhad University of Medical Sciences, Mashhad, Iran; 5 Department of Neurology, Westfälische Wilhelms-Universität Münster, Münster, Germany; 6 Epilepsy Research Center, Westfälische Wilhelms-Universität Münster, Münster, Germany

**Keywords:** Brain, Hippocampus, Intractable Epilepsy, Motor neuron, Neural stem cells

## Abstract

**Objective(s):**

Among several cell sources, adult human neural stem/progenitor cells (hNS/PCs) have been considered outstanding cells for performing mechanistic studies in in vitro and in vivo models of neurological disorders as well as for potential utility in cell-based therapeutic approaches. Previous studies addressed the isolation and culture of hNS/PCs from human neocortical and hippocampal tissues. However, little data are available on hNS/PCs obtained from the adult human amygdala.

**Materials and Methods:**

The present study explored the capacity of the amygdala harvested from resected brain tissues of patients with medically refractory epilepsy to generate neurosphere-like bodies and motor neuron-like cells.

**Results:**

Although the proliferation process was slow, a considerable amount of cells was obtained after the 3rd passage. In addition, the cells could generate motor neuron-like cells under appropriate culture conditions.

**Conclusion:**

Isolation and culture of these cells enable us to improve our knowledge of the role of the amygdala in some neurological and psychological disorders and provide a novel source for therapeutic cell transplantation.

## Introduction

In recent decades, neural stem/progenitor cells (NS/PCs) have been used in enormous basic and therapeutic investigations (1) and several sources are identified for these cells, including embryonic, fetal, and adult stem cells (2, 3). Most of these studies have focused on embryonic and fetal cells, use of which in transplantation therapy raised immunological, availability, and ethical concerns (4). In contrast, adult stem cells can be considered an outstanding cell source for autologous transplantations (5). Among different adult sources for NS/PCs, the subventricular zone (SVZ) of the lateral wall of the lateral ventricle and the subgranular zone of the hippocampus are well-identified areas as the active sources for NS/PCs (6-8). Moreover, an active neurogenesis has been identified in the subependymal zone near the hypothalamus (9). In addition to active neurogenesis in the adult mammalian brain, quiescent NS/PCs are located in different adult mammalian brain regions that can be isolated and cultured in appropriate conditions (10). 

Isolation and expansion of human neural stem/progenitor cells (hNS/PCs) from various human brain regions, including the neocortex (11), the olfactory bulb (12), the SVZ (13), the hippocampus (14, 15), and subcortical white matter (16), as well as from different brain tumors (17) have been reported. Data on the amygdala as a source for NS/PCs are scarce (11). The amygdala, a group of nuclei located in the anterior temporal lobe, is involved in several brain functions, including emotional responses, social interaction and judgments, learning and memory, and decision making (18). In addition, the amygdala is one of the brain regions involved in temporal lobe epilepsy (19). Since amygdalectomy is a part of the surgical treatment of patients with medically intractable epilepsy (20), the obtained tissue can be considered as a human source for molecular and cellular studies of brain disorders. Characterization of cellular properties of the stem cells harvested from epileptic amygdala tissue can be useful in designing reliable models of amygdala-related disorders, such as epilepsy and anxiety. Furthermore, these stem cells may be used as a source for autologous stem cell transplantation.

Generation of motor neurons from stem cells has been considered as a therapeutic choice for motor neuron diseases (21). To date, several sources were used to provide these specific neurons, including human, rodent, and primate embryonic stem cells as well as induced pluripotent stem cells (iPSCs) and murine cortical stem cells (22, 23). However, there is no report on production of motor neurons from the adult human brain. Since proliferation and differentiation capacities are two main properties of stem cells, the ability of human amygdala tissue to generate NS/PCs was evaluated in the present study. In addition, the obtained NS/PCs were exposed to a differentiation medium to test whether they can develop into motor neurons.

## Materials and Methods

***Brain tissue collection***

**Figure 1:**
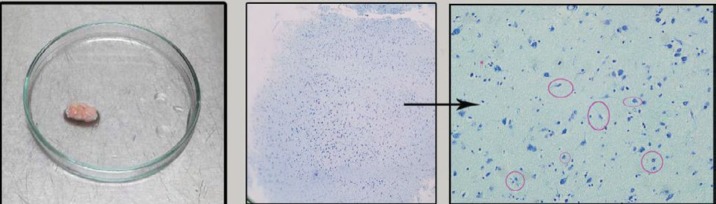
Histological verification of the amygdala tissue. Schematic design of an amygdala section and a represented example of histological confirmation based on cytoarchitecture of the amygdala complex. (morphological aspects of neurons in the amygdala are pyramidal, modified pyramidal, ovoid, and gliaform that have been labeled. types that have been labeled. Magnification of toluidine blue staining photomicrographs is 20X

All procedures were approved by the ethics committee of the Shefa Neuroscience Research Center, Tehran, Iran. Samples were collected during brain surgery for treatment of patients with medically intractable temporal lobe epilepsy. The medical history of the patients is presented in Table 1. Resected amygdala specimens from 8 patients were placed in a tube containing cold phosphate-buffered saline (PBS; Gibco, Germany) with 10% penicillin-streptomycin (Gibco, Germany) in the operating room and transported to the lab within next 5–10 min. From each individual specimen, alternate sections were used for identification of the amygdala tissues (Figure 1) (24).

***Tissue dissociation***

PBS solution was removed and the tissue was transferred to a petri dish containing 5 ml fresh PBS, washed 2 to 3 times with PBS to remove debris and associated blood vessels. Mechanical dissociation was done using a surgical knife and blood vessels were removed. For enzymatic digestion, the tissue was incubated with 1–3 ml accutase (Gibco, Germany) for 10 min at room temperature and the suspension was broken up by pipetting for 2–3 times. An equal volume of fresh medium was added to the tube to stop the enzymatic reactions. Then, the suspension was centrifuged for 5 min at 110 g at room temperature.

After disposing of the supernatant, the pellet was resuspended in 1 ml of Dulbecco’s modified Eagle’s medium/F12 (DMEM/F12; Gibco, Germany). The clumps were dissociated by gently pipetting up and down until a smooth milky single cell suspension was attained. To remove un-dissociated pieces, 10 ml of medium was added to the tube and the cell suspension was filtered through a strainer (40 μm pore size; BD Falcon) and centrifuged at 110 g for 5 min at room temperature. Next, the supernatant was discarded. The pelleted cells were then re-suspended in 1 ml of medium for cell counting.

***Cell counting and plating***

10 μl of the cell suspension was mixed with 90 μl of trypan blue 0.04% (Biomedical, USA). 10 μl of the mixture was transferred to hemocytometer in order to count the cells. The single cells were cultured in neurosphere medium, including DMEM/F12 containing 20 ng/ml EGF (Sigma, Germany), 20 ng/ml FGF2 (Sigma, Germany), 2 μg/ml heparin (Sigma, Germany)**, **1% L-glutamine (Sigma, Germany), 1% pen/strep (Gibco, Germany), 2% B27-Supplement (Gibco, Germany), and 0.5% N2-Supplement (Gibco, Germany) in non-coated flasks (~4×10^5^ cells/T-25 flask). The flasks were placed in a 37 °C incubator set at 5% CO_2_. 

***Passaging and expansion of the amygdala tissue derived spheres***

After reaching a size of about >200 µm within 2–3 weeks, the neurospheres were transferred to 15 ml tubes and centrifuged at 110 g for 5 min. The cell viability of the neurospheres reduces at the diameter larger than 200 µm (25). Then, the pellet was re-suspended in accutase (1 ml) for 10 min under the hood and later an equal volume of medium was added to the tube and pipetting was gently done 2–4 times. Following repeated centrifugation, the supernatant was discarded and the cells were re-suspended in neurosphere medium and were cultured in the appropriate size of non-coated flasks (~35×10^4^ cells/T-25 flask). The number of spheres and cells was calculated after each passage. Data are represented as the mean ± SEM.

***Differentiation***

To induce motor neuron-like cells, the neural stem-like cells were plated at 10^4^ cells/cm^2^ in 24-well plates that were pre-coated with poly-D-lysine (0.1 mg/ml in dH2O; Chemicon, USA) and laminin (5 µg/ml in dH2O; Sigma, Germany). The cells were incubated with a DMEM/F12 medium with 10% fetal bovine serum (FBS; Sigma, Germany), 1% L-glutamine, and pen/strep for 24 hr at 37 °C.

Differentiation of the cells was induced by treating the cells with DMED/F12 containing 10% FBS, 0.1% B-27 supplement, 0.5% N2-supplement, 1%L-glutamine, 200 ng/ml Sonic hedgehog (SHH, Sigma, Germany), 1 µM retinoic acid (Sigma, Germany), and 1% pen/strep, and the medium was replaced twice a week. Retinoic acid and SHH are two main factors that promote motor neuron generation during embryogenesis (26).

After 7 days, the brain-derived neurotrophic factor (BDNF; 10 ng/ml, Sigma, Germany), the glial-derived neurotrophic factor (GDNF; 10 ng/ml, Chemicon, USA), and the ciliary neurotrophic factor (CNTF; 5 ng/ml, Chemicon, USA) were added to the culture medium, which was replaced every 2 days for one week. BDNF and CNTF promote cell viability whereas GDNF increases the number of motor neurons and promotes neuritogenesis (27). Adding the abovementioned neurotrophic factors and survival-promoting compounds are necessary for developing human motor neurons from hNS/PCs (28). 

***Immunofluorescence assay***

To characterize the isolated cells, immunocyto-chemistry was performed against NS/PCs markers, nestin and Sox2; astrocyte markers, glial fibrillary acidic protein (GFAP); microglial marker, CD68; and neuronal marker, MAP-2. In addition, immunostaining for choline acetyltransferase (ChAT), insulin gene enhancer protein (Isl-1), and homeobox protein HB9 (HLXB9) was performed to evaluate the motor neuron-like cells. The cells were cultured on coverslips coated with gelatin (Sigma, Germany), fixed with 4% paraformaldehyde (Merck, USA) for 20 min, permeabilized using 0.2% Triton X-100 (Sigma, Germany) for 30 min, incubated with 5% bovine serum albumin (Sigma, Germany) for 1 hr at room temperature. The primary antibodies used overnight at 4 °C were mouse anti-nestin (1: 50 diluted in PBS; Santa Cruz), rabbit anti-Sox2 (1: 100 diluted in PBS; Santa Cruz), mouse anti-GFAP (1: 200 diluted in PBS; Millipore), rabbit anti-MAP-2 (1:500 diluted in PBS; Millipore), mouse anti-CD 68 (1:200 diluted in PBS; Abcam), rabbit anti-ChAT (1:300 diluted in PBS; Abcam), rabbit anti-Isl-1 (1:200 diluted in PBS; Abcam), and rabbit anti-HB9 (1:200 diluted in PBS; Abcam). Subsequently, the cells were washed three times with PBS and then incubated with goat anti-mouse IgG (FITC) (1:1000 diluted in PBS; Abcam) or goat anti-rabbit IgG (FITC) (1:1000 diluted in PBS; Abcam) for 1 hr at room temperature. Nuclei were stained using 4’, 6-diamidine-2-phenylindole dihydrochloride (DAPI; Sigma, Germany). The immunostained samples were photographed using a fluorescence microscope (Olympus, Japan). In control studies, the primary antibody was replaced with mouse or rabbit control IgG (Abcam, USA). There was no immunoreactivity in these controls (29).

**Figure 2:**
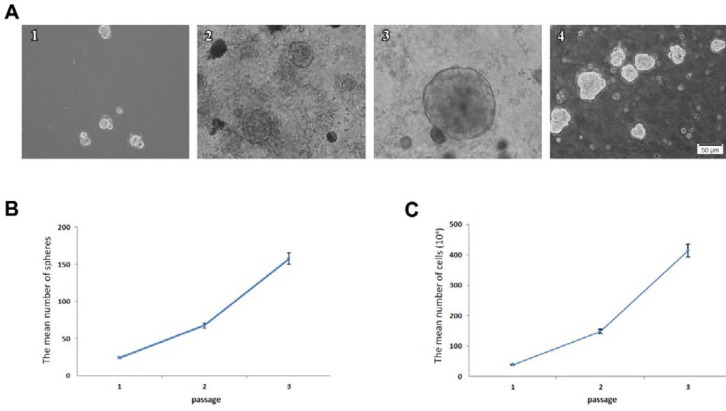
Formation of neurospheres from the epileptic human amygdala tissue. A: The neurospheres proliferated slowly 4 (A1), 7 (A2), and 14 (A3) days after primary culture of the adult human amygdala (magnification 20X). A4 represents the neurospheres at passage 3. Scale bars for all micrographs are equal to 50 µm. B: Quantification of spheres from the adult human amygdala tissue was obtained from 8 patients with refractory epilepsy during brain surgery (~150 neurospheres/amygdala tissues in passage 3). The mean number of spheres exponentially increased during three passages. C: The mean number of cells obtained after each passage. Calculation of the cell number represented a rising trend during passages

**Figure 3:**
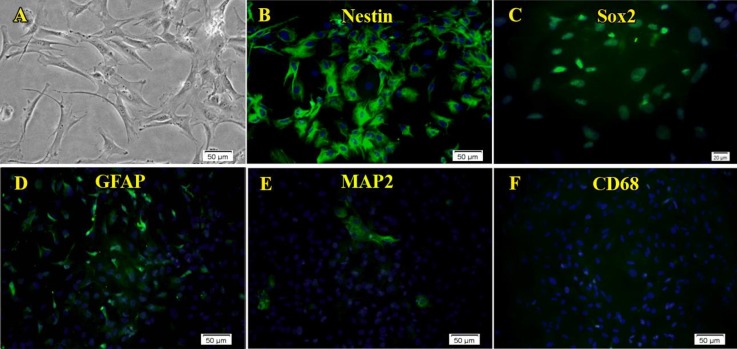
Characterization of the neural stem-like cells isolated from the epileptic human amygdala. Immunocytochemistry analysis showed that almost all of the cells isolated and cultured from the adult human amygdala by the neurosphere culture method expressed nestin (B, green) and Sox2 (C, green). In addition, the expression of GFAP was considerable (D, green). In contrast, only a few MAP-2 (E, green) or CD68 (F) positive cells were seen. A is a phase contrast micrograph of adherent neural stem-like cells. Nuclei are seen in blue

**Figure 4:**
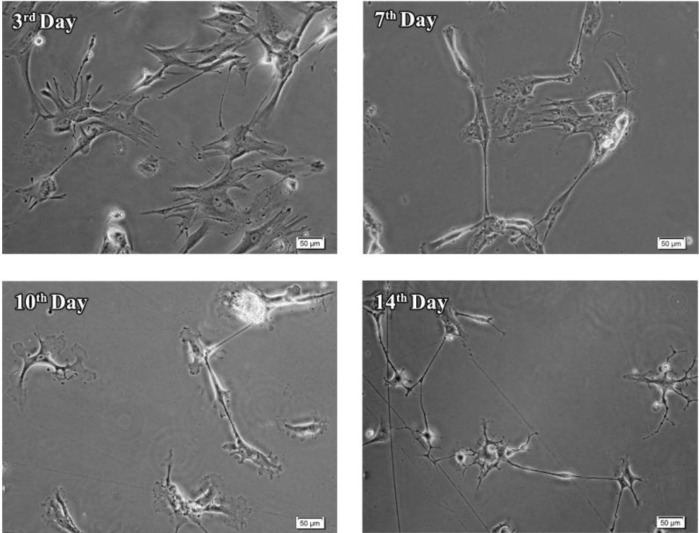
Morphological changes during differentiation of neural stem-like cells. Neural stem-like cells obtained from the epileptic human amygdala differentiated during 14 days exposure to differentiation medium

**Figure 5:**
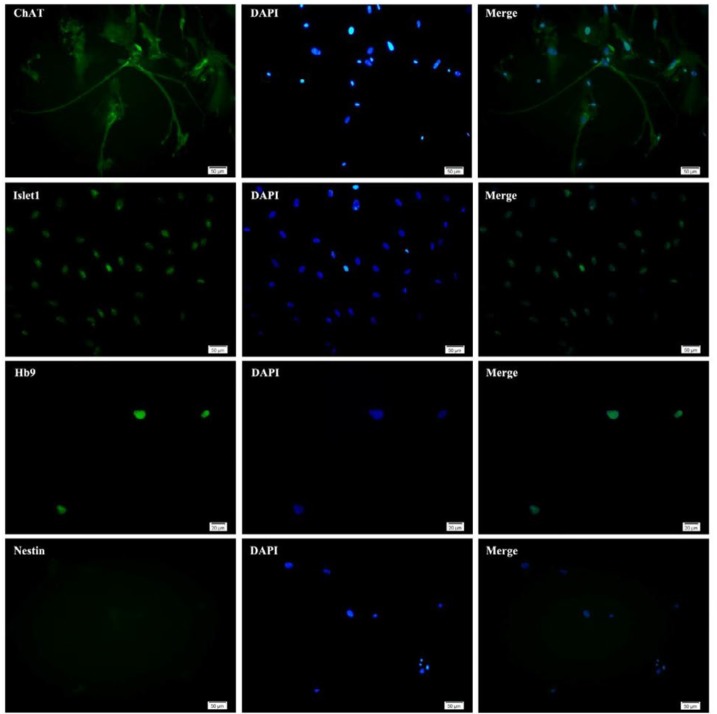
Generation of motor neurons from neural stem-like cells from human amygdala obtained during epilepsy surgery. The cells were evaluated for motor neuron differentiation. Generated neurons expressed motor neuron-specific markers ChAT, Isl-1, and HB9 but not neural stem cells marker, nestin

**Table 1:** Medical history of patients. The tissues were obtained from 8 patients with refractory epilepsy undergone amygdalohippocampectomy

**Case**	**Gender**	**Age** **(year)**	**Age at the onset of the first seizure (year)**	**Seizure frequency**	**Drug history**	**Histology and imaging**	**Seizures**
1	Female	24	1	5-10 weekly	LEV, LTG	Sclerosis	GS
2	Male	35	32	1-2 monthly	CBZ, VPA	Sclerosis	GS
3	Female	39	4	2-3 monthly	LEV, CBZ, LTG	Sclerosis	GS
4	Female	37	7	2-3 daily	CBZ, LTG, PRM	Sclerosis	GS
5	Female	30	18	1-2 weekly	LEV, TOP	Sclerosis	GS
6	Male	42	19	1 weekly	LEV, OCBZ	Sclerosis	GS
7	Male	13	2	7-8 daily	VPA, OCBZ, LEV	Sclerosis + occipital lobe vascular abnormality	PS
8	Male	30	5	3-4 monthly	LEV,VPA, CBZ, TOP	Sclerosis	PS

CBZ: carbamazepine; GS: generalized seizures; LEV: levetiracetam; LTG: lamotrigin; OCBZ: oxcarbazepine; PS: partial seizure; TOP: Topiramate; VPA: valproate

## Results

***Neurosphere assay***

Neural stem-like cells proliferated after the initial tissue culture in the neurosphere medium. The free-floating neurospheres were observed four days after the primary culture of the amygdala tissue (Figure 2 A1). The diameter of most neurospheres reached about 200 µm after 2 weeks of the primary culture (Figure 2, A2 and 3). At this time, the neurospheres were ready for the passaging procedure. Secondary neurospheres were formed 5 to 8 days after the first passage. The number of neurospheres increased after each passage (Figure 2 A4). 

***Proliferation assay***

Before each passage, eight fields were chosen randomly and the number of spheres was counted by 10X objective in each flask. An increasing trend was observed in the mean number of neurospheres after each passage. However, the process of cell proliferation was slow. In the first passage (2 weeks after primary culture), the mean number of primary neurospheres was 24.3 ± 0.5. The mean number of neurospheres increased to 67.6 ± 0.3 after 12 days and to 157.6 ± 9.9 after 15 days in the second and third passages, respectively (Figure 2 B). In line with neurosphere proliferation, the mean number of cells also enhanced after each passage (Figure 2 C). 

***Characterization of the obtained cells***

After the third passage, characteristic features of cells were evaluated using immunocytochemistry. The majority of isolated cells expressed progenitor NS/PCs markers, nestin and Sox2. Moreover, mature neuron marker MAP-2, as well as astrocyte marker GFAP, were expressed in a considerable number of cells. The cells showed little or no immunoreactivity for CD68 (Figure 3).

***Differentiation assay***

To evaluate the differentiation capacity, the potential of the obtained cells to differentiate into motor neurons was investigated. As indicated in Figure 4, the morphology of cells changed during the first week of differentiation, in which the cells were exposed to SHH. These cells displayed neurite-like outgrowth at the periphery of their cell bodies. Adding trophic factors in the second week promoted the differentiation of the cells. Morphological analysis at day 14 revealed maturation of some motor neurons with elongated processes (Figure 4). Immunofluorescence studies indicated that the cells expressed motor neuron markers, ChAT, Isl-1, and HB9, 14 days after exposure to the differentiation medium. In contrast, the expression of the neural stem cell marker, nestin was very low in these cells (Figure 5).

## Discussion

The present data revealed the potential of the resected amygdala tissues during epilepsy surgery to generate neurospheres. Furthermore, our study revealed that these neurospheres under definite conditions could be differentiated into motor neuron-like cells. To date, only a few investigations have tested the isolation of hNS/PCs (11,16). We addressed a reproducible method for culturing the neurosphere-like bodies from the adult human amygdala. 

The majority of the cells isolated from the adult human amygdala in the present study expressed nestin and Sox2, two main markers of NS/PCs (30, 31), whereas GFAP, a marker of astrocytes (32), was expressed in a considerable number of cells. Considering the fact that the expression of MAP-2 and CD68 in the cells were low, it can be concluded that these cells maintained their stemness capacity in the neurosphere medium. The process of cell proliferation in this specific type of human brain tissue is slow. It is worth pointing out that any excess shaking of culture flasks during the first four days of primary culture decreases the quality of neurosphere culture and may affect cell proliferation.

The results of the present study revealed that the stem cells harvested from human amygdala could differentiate into motor neurons using SHH and retinoic acid. Previous studies reported the production of motor neurons from the other sources. It has been reported that motor neurons can be generated from iPSCs derived from a patient with amyotrophic lateral sclerosis using an agonist of the SHH signaling pathway and retinoic acid (21). Furthermore, motor neurons have been produced from embryonic stem cells. They reported that these stem cells could differentiate into motor neurons by developmentally relevant signaling factors (33). Hester and colleagues revealed that the generation of functional motor neurons from iPSCs is a prolonged process that required about 60 days. Using motor neuron-inducing transcription factors, they could reprogram the stem cells and reduce the differentiation time to 30 days (22). Induced pluripotent stem cell-derived motor neurons have been suggested for the treatment of amyotrophic lateral sclerosis and motor neuron diseases (34).

Several million people worldwide suffer from medically intractable epilepsy and the risk of seizures is associated with marked mortality and co-morbidity (35). Animal models and *in vitro* human-derived iPSC models improved our understanding of various aspects of epilepsy and suggested the importance of cell therapy in the treatment of intractable epilepsy (36). Autologous stem cell therapy is a therapeutic option that is gaining ground for the treatment of different neurological and psychiatric disorders and can be used for the treatment of refractory epilepsy (37, 38). Since the cells in the present study were isolated from epileptic patients, characterization of the obtained stem-like cells is very important. Determination of the electrophysiological properties, as well as the pattern of gene expression, can be helpful in future applications of these motor neurons. If hNS/PCs obtained from epileptic surgery have epileptic activity, they could be utilized for creation of cell-based models of intractable epilepsy and provide valuable information about cellular and molecular mechanisms of epilepsy. If properties of these stem-like cells are not similar to those of epileptic cells, they could be considered an outstanding source in autologous cell-based therapeutic strategies. Furthermore, hNS/PCs obtained from human amygdala may contribute to our understanding of pathophysiological mechanisms of other amygdala-related disorders, such as anxiety (39, 40).

In conclusion, human amygdala tissue not only provides a valuable source for hNS/PCs but also can be differentiated into motor neurons. Further characterization of the generated cells is required to use them for mechanistic studies or therapeutic applications.

## Conflicts of Interest

The authors announce no competing financial interests.
